# CircRPPH1 promotes hepatocellular carcinoma progression through inhibiting PPARα transcription and accelerating its ubiquitination and degradation

**DOI:** 10.1007/s00018-026-06211-3

**Published:** 2026-04-30

**Authors:** Wenxiu Ru, Yu Lu, Lunbiao Gan, Siyu Yao, Huanhuan Wan, Fengze Nie, Sijia Di, Yujing Guo, Qian Huang, Sha Yin, Jiaqi Ning, Fa Yang, Weijun Qin, Weihong Wen

**Affiliations:** 1 School of Life Sciences and Technology, Northwestern Polytechnical University, Xi’an, 710072 China; 2 Department of Urology, Xijing Hospital, Fourth Military Medical University, Xi’an, 710032 China

**Keywords:** Hepatocellular carcinoma (HCC), circRPPH1, PPARα, miR-7845-5p, SOX4, USP1

## Abstract

**Background:**

Hepatocellular carcinoma (HCC) is a prominent cause of cancer-related mortality globally. It is urgently necessary to elucidate the pathogenesis of HCC and develop novel therapeutic strategies. Although certain circular RNAs (circRNAs) act as oncogenic drivers in HCC progression, the underlying mechanisms remain poorly understood.

**Methods:**

CircRNA-miRNA-mRNA network was established to screen potential circRNAs that were associated with HCC progression. CCK8, colony formation, EdU staining, wound healing, transwell assay and xenograft models were implemented to investigate the role of circRPPH1 in HCC progression. RNA-seq, luciferase reporter assays, Turbo-ID, mass spectrometry, co-immunoprecipitation and RNA-binding protein immunoprecipitation assays and rescue experiments were employed to determine the underlying molecular mechanism.

**Results:**

CircRPPH1 was significantly upregulated in HCC tissues and it promoted HCC progression both in vitro and in vivo. The elevated circRPPH1 expression in HCC was due to its increased stability, which was mediated by m^6^A methylation. CircRPPH1 could suppress the level of PPARα to promote HCC progression. Mechanistically, we found that circRPPH1 sponged miR-7845-5p to upregulate the expression of SOX4, which inhibited the transcription of PPARα. CircRPPH1 could also competitively bind with USP1, resulting in increased ubiquitination and degradation of PPARα. We also demonstrated that circRPPH1-ASOs could effectively inhibit HCC growth in vivo.

**Conclusions:**

CircRPPH1 is a tumor-promoting circRNA, which inhibits PPARα transcription through miR-7845-5p/SOX4 axis and accelerates its ubiquitination and degradation through competitively binding with USP1. These findings suggest that circRPPH1 may serve as a promising therapeutic target for HCC treatment.

**Supplementary Information:**

The online version contains supplementary material available at 10.1007/s00018-026-06211-3.

## Introduction

Hepatocellular carcinoma (HCC) is the most predominant type of primary liver cancer, representing 90% of all liver malignancies. Notably, HCC ranks as the third highest cause of cancer-related mortality, and its global incidence and mortality rates are still rapidly increasing [1]. Although treatment strategies for HCC have significantly progressed in the last decade, the prognosis and survival rate for HCC are still remain unsatisfactory [2]. Thus, it is urgently needed to identify the key molecules that promote HCC progression and elucidate the underlying mechanisms.

Epigenetic mechanisms play an important role in HCC progression. Recently, this link has been highlighted by circular RNA (circRNA) bringing about changes [3]. CircRNA is a class of non-coding RNAs characterized by a covalently closed-loop structure, stably existing in tissues and cells. CircRNAs were originally found to exert their function through serving as a miRNA sponge and interacting with some proteins [4]. In addition, certain circRNA can be translated into functional peptides [5]. Several circRNAs that are dysregulated in HCC have been identified, which could be potential diagnostic and therapeutic targets. Additionally, mounting evidence has shown that some circRNAs play critical regulatory roles in HCC progression, where they exhibit tumor-promoting or tumor-suppressive effects through various mechanisms [6]. For instance, circASH2 was found to induce the liquid–liquid phase separation (LLPS) of YBX1 to enhance the decay of TPM4 transcripts and thus inhibit HCC metastasis, whereas circPIAS1 was shown to promote HCC progression through inhibiting ferroptosis via the miR-455-3p/NUPR1/FTH1 axis [7, 8]. Yet, studies on tumor-promoting or tumor-suppressive circRNAs in HCC are still limited, and their detailed mechanisms still need to be elucidated.

N6-methyladenosine (m^6^A) is one of the most common post-transcriptional modifications of RNA, participating in various aspects of RNA homeostasis [9]. Importantly, m^6^A has been found to be involved in the regulation of biogenesis, stability, translation, and nuclear export of circRNAs [10]. Further exploration of the molecular links between m^6^A modification and circRNA characteristics is essential for the understanding of the dysregulated level of circRNAs in certain cancer types.

Peroxisome proliferator-activated receptor alpha (PPARα) is highly expressed in liver and plays a major role in the metabolism of fatty acids and in lipid transport [2]. Disruption of PPARα expression in hepatocytes resulted in impaired fatty acid oxidation (FAO) and aggravation of hepatic steatosis [11]. Recently, dysregulation of PPARα has been found to be closely related to the development and occurrence of HCC [2]. Some studies have found that PPARα exhibited tumor-suppressive functions, and its expression was decreased in HCC samples [12–15]. Several noncoding RNAs, including hsa-miR-21-5p, miR-708 and hsa_circ_0110102, have been found to regulate the PPARα signaling pathway to promote HCC progression [14–16]. However, to date, the detailed mechanisms how certain circRNAs regulate PPARα are still poorly understood. Therefore, due to the crucial role of PPARα in HCC progression the identification of certain circRNAs that regulate PPARα may help to discover novel therapeutic targets for HCC treatment.

In this work, through the analysis of an HCC-related circRNA-miRNA-mRNA network, we found that circRPPH1 was significantly upregulated in HCC tissues and it could promote HCC progression both in vitro and in vivo. Notably, the elevated expression of circRPPH1 is mediated by METTL3-dependent m^6^A methylation. We also found that circRPPH1 could promote the growth and metastasis of HCC by inhibiting PPARα transcription through the miR-7845-5p/SOX4 axis and accelerating the degradation of PPARα through competitively binding with USP1, which is a deubiquitinase that mediates PPARα stabilization. Furthermore, treatment with circRPPH1-ASO significantly inhibit HCC growth in vivo, indicating that circRPPH1 could serve as a potential therapeutic target for HCC treatment.

## Materials and methods

### Tissue samples

To compare circRPPH1 levels in HCC tissues, 9 pairs of fresh HCC and adjacent normal liver tissues were obtained from surgical specimens after hepatectomy at Xijing Hospital (Xi’an, China). The use of the specimens was approved by the Institutional Ethics Committee of the Northwestern Polytechnical University, and informed consent was obtained from all patients.

### Cell lines and functional assays in vitro

Human HCC cell lines (Huh7, HepG2, PLC/PRF/5, Hep3B, HCC-LM3, MHCC97-L and SK-Hep1) and HEK293T were purchased from Pricella (Wuhan, China). Cells were cultured in DMEM or MEM supplemented with 10% FBS (Biological Industries) and 1% penicillin/streptomycin (Solarbio, China) at 5% CO2 humidified incubator at 37 °C. The cells were systematically screened for mycoplasma presence, interspecies contamination, and genetic authenticity before being used in our study.

CCK8, colony formation, EdU, transwell and wound healing assays were described in the supplementary materials and methods.

### Dot-blot assay

Diluted RNAs were denatured (65 °C, 10 min) and spotted onto an N^+^ membrane (Biotopped, Amersham). After UV cross-linking for 10 min and blocking with 5% BSA, membranes were incubated with anti-m^6^A antibody (Merck, Germany, RRID: AB_3674612) overnight at 4 °C, washed with TBST, and incubated with a secondary antibody. Signals were visualized using a chemiluminescence system (Vazyme, China). For methylene blue staining, membranes were incubated in 0.3 M sodium acetate (pH 5.2) containing 0.02% methylene blue.

### RNA fluorescence in situ hybridization (FISH)

The specific probe targeting the splicing site of circRPPH1 was designed and synthesized by Servicebio (China). Cells were seeded into the 6-well plates containing cover glasses, then were fixed with in situ hybridization fixative (Servicebio, China). After permeabilization with 0.1% Triton X-100, the cells were exposed to FAM-labelled circRPPH1 probes overnight at 37℃. Cell nuclei were stained with DAPI (Solarbio, China). Confocal laser scanning microscope was used to acquire the images (OLYMPUS, Japan).

### Plasmid construction, oligonucleotide synthesis and transfection

The full-length sequence of the circRPPH1 was cloned into the pCDNA-2.1 vector to achieve overexpression. The expression plasmid of pCD25-circRPPH1-MS2bs was synthesized by GENESEED (Guangzhou, China). The coding sequences of METTL3, Myc-tagged PPARα, Flag-tagged SOX4, and Flag-tagged USP1 were cloned into the pcDNA3.1(+) vector. Small-interfering RNA targeting circRPPH1, METTL3, METTL14, YTHDC1, IGF2BP1, IGF2BP3, SOX4 and the oligonucleotides of the miR-7845-5p mimic were synthesized by General Bio (Anhui, China). The sequences of the siRNAs and miRNA mimics used are listed in Supplementary Table S2. Plasmids and oligonucleotides were transfected using Lipofectamine 2000 (Invitrogen), according to the manufacturer’s instructions.

### Lentivirus packaging and infection

A lentiviral vector carrying the full length circRPPH1 was synthesized by HanBio (Shanghai, China). Lentiviruses for TurboID-MS2 were produced by co-transfecting constructed plasmids pLVX-Puro and the packaging plasmids psPAX2 and pMD2.G into 293T cells for 48 h. Culture supernatants containing lentivirus were collected, filtered, and concentrated. Huh7 or Hep3B cells were infected with lentivirus in the presence of 6 µg/mL polybrene (Beyotime, China). After 72 h, the infected cells were screened with 4 µg/mL puromycin (Beyotime, China) for 7 days. The stably transfected cells were established when the cells became resistant to puromycin.

### RNA immunoprecipitation assay (RIP)

The RIP assay was performed using RIP-Assay Kit (MBL, Japan) according to the manufacturer’s instructions. In brief, the protein A/G magnetic beads (Thermo, Waltham) were incubated with specific antibodies and IgG. Subsequently, HCC cells were lysed using the RIP lysis buffer and incubated overnight with protein A/G magnetic beads coated with antibodies. After washing, the RNA was isolated from mRNPs and analyzed by qRT-PCR assay.

### Turbo-ID proximity labeling assay and mass spectrometry

HCC cells that had been stably transfected with TurboID-MS2 were cultured in 100 µM biotin at 37 °C for 10 min. Subsequently, the cells were placed on ice for 15–30 s, followed by three washes with ice-cold PBS. Cells were lysed using the protein extraction reagent RIPA containing 1 mM PMSF and protease inhibitor (MCE, America). Cell lysates were incubated with M-280 Streptavidin Dynabeads (Thermo, Waltham) at 4℃ for 2 h and washed with PBS. The biotinylated proteins were eluted from the dynabeads for mass spectrometry or Western blot analysis. The mass spectrometry was performed by BIOPROFILE (Shanghai, China).

### ChIP-qPCR

Chromatin immunoprecipitation (ChIP) assay was conducted using Sonication ChIP Kit (ABclona, China) following the manufacturer’s instructions. Briefly, Huh7 cells transfected with the pCMV5-SOX4-Flag plasmid were crosslinked with 1% paraformaldehyde for 15 min and subsequently lysed. The chromatin was sonicated into fragments of 300–500 bp. The lysates were then incubated with 5 µg of anti-Flag (AE092, ABclona), anti-IgG (2729, CST) or anti-Histone H3 (RM20711, ABclona). The complexes were captured using Protein A/G Magnetic Beads (HY-K0202, MCE) at 4 °C for 3 h. After that, the precipitated DNA was de-cross-linked and purified using FastPure Gel DNA Extraction Mini Kit (Vazyme, China). Finally, the purified DNA was subjected to qPCR analysis using 5% input as a control. The sequences of primers used are listed in Supplementary Table S1.

### RNA sequencing

Total RNA was extracted from control and circRPPH1-overexpressing Huh7 cells and sequenced by NovelBio (Shanghai, China). Biological pathway analysis was performed using clusterProfiler.

### Animal study

All of the experiments involving animals were approved by the Laboratory Animal Welfare and Ethics Committee of the Northwestern Polytechnical University. The male BALB/c nude mice aged between 4 and 6 weeks were purchased from the Animal Center of the Fourth Military Medical University (Xi’an, China). To establish a subcutaneous tumor model, Huh7 cells (1 × 107) were resuspended in medium containing 25% Matrigel (Solarbio, China), and then were subcutaneously injected into the axilla of mice. To establish an orthotopic tumor model, the anesthetized mice were subjected to surgery to fully expose the liver, and were injected with Huh7 cells expressing luciferase suspended in 50% matrigel. For treatment experiments in vivo, ASOs specifically targeting circRPPH1 were designed by IBSBIO (Shanghai, China). The subcutaneous tumor model was established as previously described, and ASO-Ctrl (5 nmol) or circRPPH1-ASO (5 nmol) were intratumorally injected every three days for three consecutive weeks, starting after the tumor size reached 100 mm³. Tumor growth was monitored every 3 days during the animal experiment. After 4 weeks, the mice were euthanized, and the liver tissues and subcutaneous tumors were harvested for subsequent analysis.

Additional information on materials and methods is provided in the supplementary materials and methods.

### Statistical analysis

All experiments were performed at least three times and the data were presented as mean ± SD. Statistical analyses were determined by the unpaired Student’s T test or a two-way ANOVA using GraphPad Prism 8.0 (SCR_002798). Statistical significance was indicated as *p < 0.05, **p < 0.01, ***p < 0.001.

## Results

### CircRPPH1 is upregulated in HCC

**Fig. 1:**
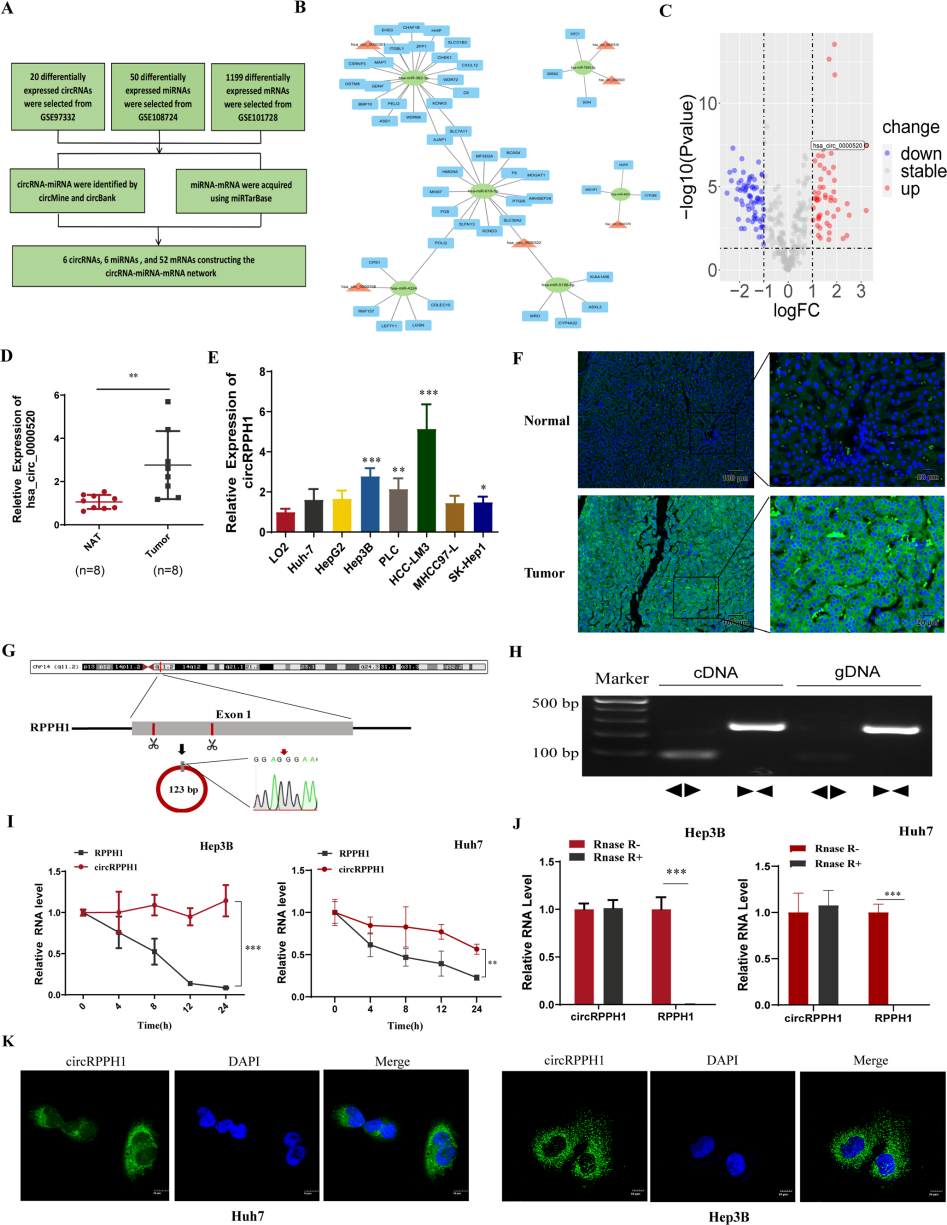
Identification and characterization of circRPPH1 in HCC. **A** Schematic diagram of data processing. **B** Global view of the circRNA-miRNA-mRNA network in HCC. **C** Volcano plot of differentially expressed circRNAs in HCC tissues and adjacent normal ones. **D** Validation of circRPPH1 expression in 9 paired HCC and paired NATs using qRT-PCR. **E** Analysis of circRPPH1 expression in HCC cell lines using qRT-PCR. **F** Representative images for the circRPPH1 expression in HCC tissues and paired NATs using FISH (scale bar, 10 μm). **G** Schematic illustration of the genomic location and back splicing of circRPPH1. Validation of back-splicing junction of circRPPH1 by Sanger sequencing. **H** RT-PCR analysis of circRPPH1 and RPPH1 mRNA using divergent and convergent primers. **I**, **J** Analysis the stability of circRPPH1 and RPPH1 after treatment with actinomycin D or RNase R. **K** Analysis of the subcellular localization of circRPPH1 using FISH. *P < 0.05, **P < 0.01 and ***P < 0.001

We constructed a circRNA-miRNA-mRNA network and systematically analyzed potential circRNAs associated with hepatocellular carcinoma (HCC) progression. After processing (Fig. 1A), 6 co-expressed circRNAs, 6 miRNAs, and 52 mRNAs were identified as key network components (Fig. 1B). Subsequent KEGG pathway analysis demonstrated significant enrichment of these network-associated RNAs in cellular metabolic processes (Fig. S1A). Among these candidate circRNAs, we noticed that hsa_circ_0000520 was significantly upregulated in HCC tissues (Fig. 1C and Fig. S1B). Previous studies have indicated that RPPH1 gene-derived circRNAs exert tumor-promoting effects in multiple malignancies, including hsa_circRNA_000166 in breast cancer, hsa_circ_0000512 in glioblastoma, and hsa_circ_0000515 in non-small cell lung cancer [17–19]. Despite these established oncogenic roles of its genetic counterparts, the functional significance of hsa_circ_0000520 (named as circRPPH1 in this study) in HCC progression remains unexplored. Consequently, hsa_circ_0000520 was selected for in-depth investigation.

To validate the expression of circRPPH1 in HCC, we analyzed its levels using qRT-PCR and fluorescence in situ hybridization (FISH). As shown in the result, the level of circRPPH1 was substantially increased in HCC tissues and cell lines compared to adjacent non-cancerous tissues and Lo2 cells (Fig. 1D-F). CircRPPH1 is derived from exon 1 of the host gene RPPH1 through back-splicing, which was preliminarily confirmed by Sanger sequencing (Fig. 1G). RT-PCR with convergent and divergent primers revealed the closed-loop structure of circRPPH1 (Fig. 1H). Additionally, circRPPH1 exhibited greater resistance to actinomycin D and RNase R treatment compared to its linear form (Fig. 1I, J). To assess the subcellular distribution of circRPPH1, FISH assays indicated that circRPPH1 was observed in both the nucleus and cytoplasm of Hep3B and Huh7 cells, while the cytoplasmic distribution was dominant (Fig. 1K). Collectively, these data indicate that circRPPH1 is upregulated in HCC and suggest its potential involvement in HCC progression.

### Increased circRPPH1 expression in HCC is due to m^6^A methylation

**Fig. 2:**
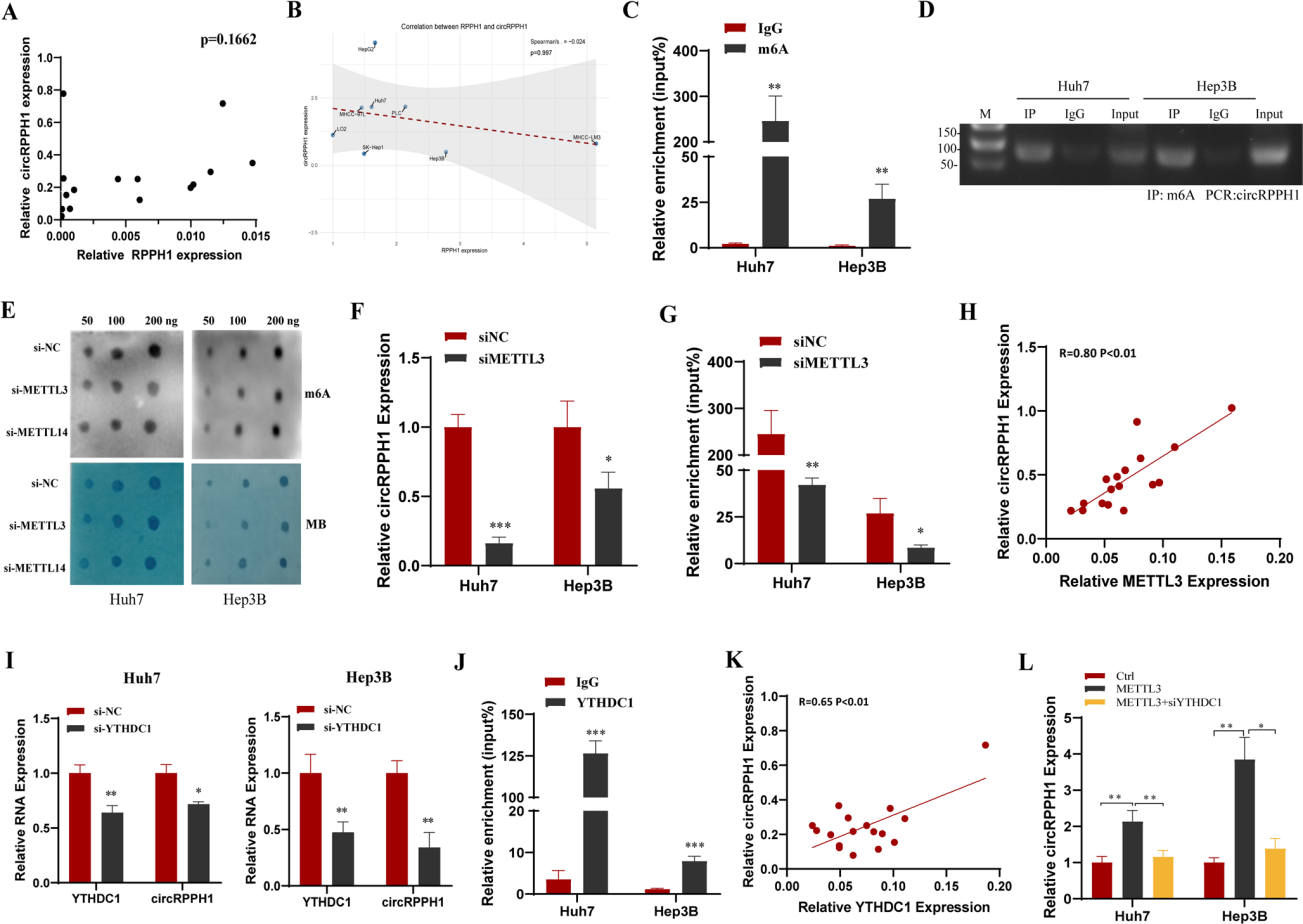
METTL3-mediated m^6^A modification enhanced the stability of circRPPH1 in HCC. **A** The correlation analysis of circRPPH1 and RPPH1 mRNA in paired samples of HCC. **B** The correlation analysis of circRPPH1 and RPPH1 mRNA in HCC cell lines. **C** MeRIP-qPCR analysis of the abundance of m6A-modified circRPPH1 in Huh7 and Hep3B. **D** MeRIP-PCR assay to detect circRPPH1 in Huh7 and Hep3B. **E** Dot plot assay to detect the m6A level in HCC cells after treatment with siMETTL3 or siMETTL14. **F** The expression of circRPPH1 in siMETTL3 HCC cells using qRT-PCR. **G** MeRIP-qPCR analysis of the relative abundance of m6A-modified circRPPH1 in siMETTL3 HCC cells. H The correlation analysis of circRPPH1 and METTL3 in paired samples of HCC. I The expression of YTHDC1 and circRPPH1 in siYTHDC1 HCC cells using qRT-PCR. **J** RIP assay to detect the binding of circRPPH1 and YTHDC1 in Huh7 and Hep3B. **K** The correlation analysis of circRPPH1 and YTHDC in paired samples of HCC. **L** The expression of circRPPH1 in HCC cells after co-transfection with METTL3 overexpression vector and siYTHDC1 using qRT-PCR. *P < 0.05, **P < 0.01 and ***P < 0.001

We next explored the detailed mechanisms governing circRPPH1 overexpression in HCC. Initially, we found that the RPPH1 mRNA levels exhibited no significant difference between HCC and normal tissues (Fig. S2A). Furthermore, there was no correlation between the levels of circRPPH1 and linear RPPH1 in HCC patient tissues and cell lines (Fig. 2A, B). It has been reported that m^6^A modification can functionally regulate the biogenesis of circRNAs [10]. To investigate whether circRPPH1 undergoes m^6^A modification, we predicted the potential m^6^A sites in circRPPH1 using SRAMP and identified one highly confident m^6^A site (Fig. S2B). Additionally, MeRIP assays revealed that circRPPH1 was significantly enriched by anti-m^6^A antibodies compared to anti-IgG controls (Fig. 2C, D). We then examined which m^6^A writers (METTL3, METTL14) contribute to the m^6^A modification of circRPPH1. By silencing METTL3 and METTL14 (Fig. S2C), we found that only METTL3 knockdown resulted in a notable reduction in m^6^A levels and circRPPH1 expression in HCC cells (Fig. 2E, F and Fig. S2D). Notably, METTL3 silencing decreased the levels of m^6^A modification in circRPPH1 (Fig. 2G). Additionally, METTL3 is overexpressed in HCC tissues (Fig. S2E), and the level of circRPPH1 is significantly correlated with METTL3 expression (Fig. 2H).

Given that m^6^A readers are responsible for the fate of m^6^A-modified RNA, we examined several readers that have been reported to affect circRNA activity, including YTHDC1, IGF2BP1 and IGF2BP3 [9, 20, 21]. The results showed that YTHDC1 inhibition led to a decrease in circRPPH1 expression (Fig. 2I), whereas no effect was observed upon IGF2BP3 and IGF2BP1 knockdown (Fig. S2F, G). RIP assays confirmed that YTHDC1 could enrich circRPPH1 RNA fragments (Fig. 2J), and a positive correlation was observed between YTHDC1 and circRPPH1 in HCC (Fig. 2K and Fig. S2H). Moreover, YTHDC1 silencing blocked the stimulatory effect of METTL3 on circRPPH1 expression (Fig. 2L). All these results suggest that the METTL3-mediated m^6^A methylation contributes to the elevated expression of circRPPH1 in HCC through YTHDC1.

### CircRPPH1 promotes the proliferation and migration of HCC cells

**Fig. 3:**
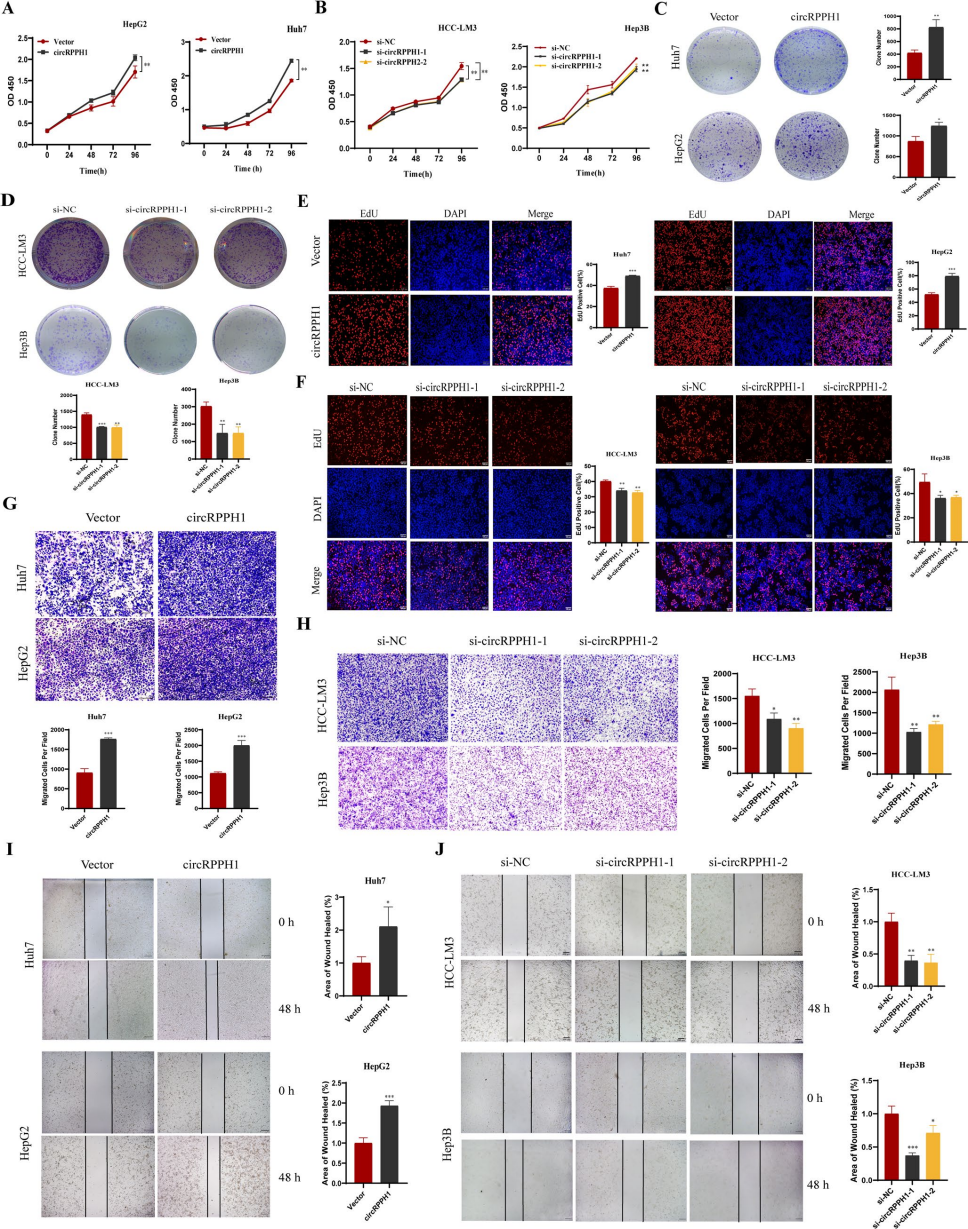
CircRPPH1 promotes HCC proliferation and migration *in vitro*. **A**-**F** Proliferation ability of HCC cells was detected by CCK8, colony formation assay and EdU assay (scale bar, 100 μm) after knockdown or overexpression of circRPPH1. (**G**-**J**) Migration ability of HCC cells was detected by transwell assay (scale bar, 100 μm) and wound healing assay (scale bar, 200 μm) after knockdown or overexpression of circRPPH1. *P < 0.05, **P < 0.01 and ***P < 0.001

To determine the biological roles of circRPPH1 in HCC progression, we designed two siRNAs specifically targeting the back-splicing site of circRPPH1 as well as an overexpression vector, to knockdown or overexpress circRPPH1, respectively. The efficiency of circRPPH1 overexpression was verified using qRT-PCR in HepG2 and Huh7 cells, while the knockdown efficacy of circRPPH1 was confirmed in HCC-LM3 and Hep3B (Fig. S3A, B). Notably, the knockdown of circRPPH1 had no significant impact on the linear RPPH1 (Fig. S3B). As demonstrated by the CCK-8 and colony formation assays, knockdown of circRPPH1 significantly inhibited the proliferation of HCC cells, whereas circRPPH1 overexpression promoted HCC cell proliferation (Fig. 3A-D). The EdU results also showed that HCC cells with circRPPH1 silencing exhibited attenuated DNA replication activity, whereas circRPPH1 overexpression led to enhanced DNA replication (Fig. 3E, F). In addition, we evaluated the effect of circRPPH1 on migratory ability of HCC cells using Transwell and wound healing assays. The results demonstrated that the migration of cells was enhanced by circRPPH1 overexpression and inhibited by circRPPH1 silencing (Fig. 3G-J). These findings collectively suggest that circRPPH1 exerts oncogenic functions in HCC progression.

### CircRPPH1 promotes HCC progression by inhibiting PPARα signaling

**Fig. 4:**
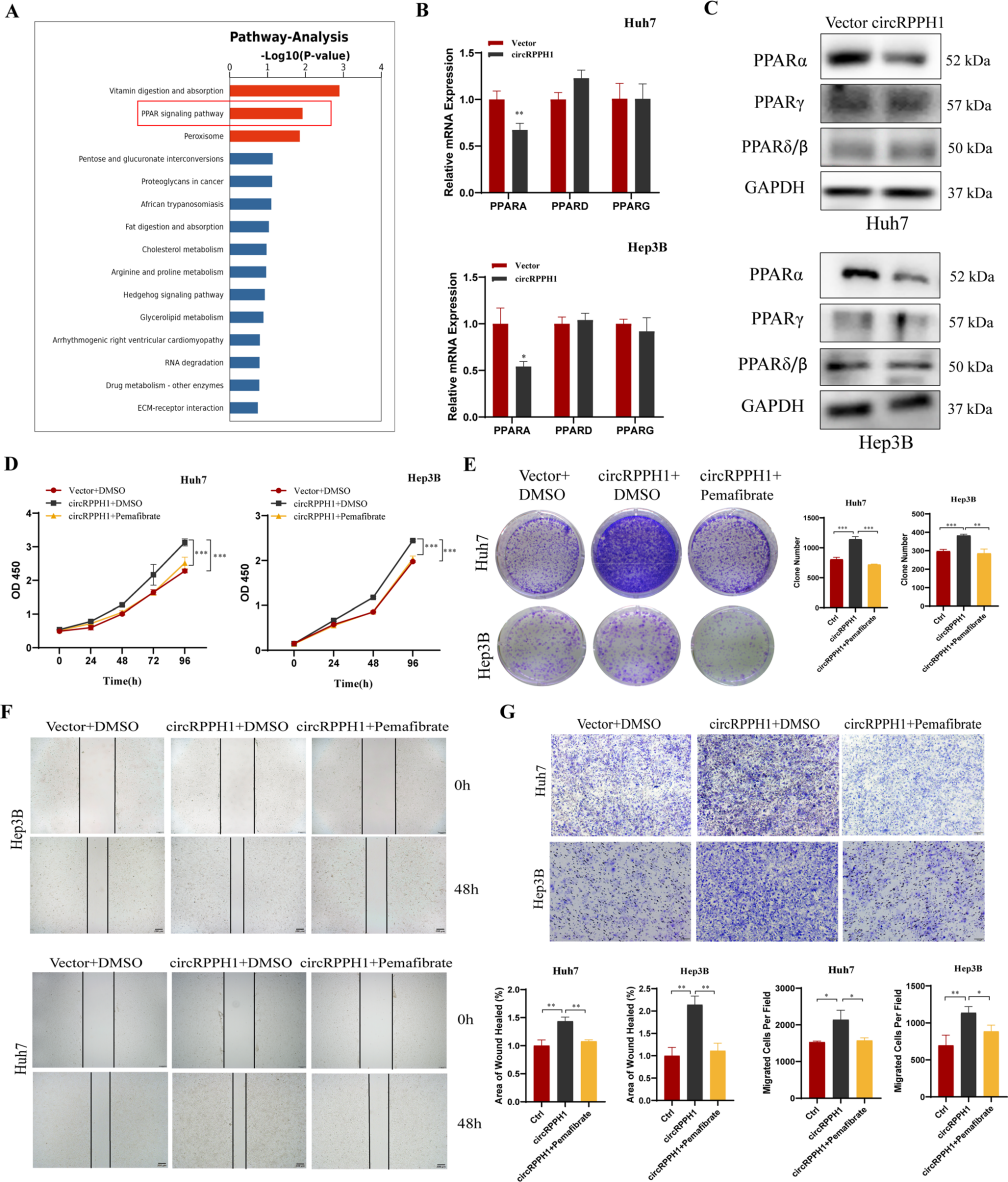
CircRPPH1 upregulates PPARα expression in HCC cells. **A** KEGG pathway analysis of differentially expressed genes. **B**, **C** The expression level of PPARα, PPARγ and PPARδ/β in HCC cells overexpressing circRPPH1 using qRT-PCR and Western blot. **D**, **E** Proliferation ability was detected by CCK8 and colony formation assay in HCC cells overexpressing circRPPH1 and treated with pemafibrate. **F**-**G** Migration ability was detected by transwell assay (scale bar, 100 μm) and wound healing assay (scale bar, 200 μm) in HCC cells overexpressing circRPPH1 and treated with pemafibrate. *P < 0.05, **P < 0.01 and ***P < 0.001

We performed RNA-seq using circRPPH1-overexpressing Huh7 cells or control cells to identify the downstream pathways of circRPPH1-triggered tumor promotion. KEGG analysis revealed that circRPPH1 is involved in the PPAR signaling pathway, as well as fat digestion and cholesterol metabolism (Fig. 4A). Notably, as previously demonstrated in our results, the co-expressed RNAs within the circRNA-miRNA-mRNA network were also implicated in the PPAR signaling pathway (Fig. S1B), suggesting that the PPAR signaling pathway may be a key mechanism underlying circRPPH1-driven HCC progression. Peroxisome proliferator-activated receptors (PPARs) include three isoforms: PPARα (PPARA), PPARβ/δ (PPARD), and PPARγ (PPARG), which play critical roles in regulating energy homeostasis and cell fate. Recent studies have elucidated that the PPAR signaling pathway contributes to HCC initiation and progression by modulating lipid homeostasis [2]. HCC cells overexpressing circRPPH1 exhibited a significant reduction in PPARα expression at both the mRNA and protein levels, whereas PPARβ/δ and PPARγ expression remained relatively unchanged (Fig. 4B, C).

Subsequently, we analyzed the expression of key lipometabolic enzymes, including ACOX1, CPT1A, CPT2, ECHS1, as well as key lipogenic enzymes, such as ACACA, ACLY, FASN, SCD1, in HCC cells overexpressing circRPPH1. The results indicated that circRPPH1 overexpression downregulated the expression of lipometabolic enzymes, while modestly upregulating lipogenic enzymes (Fig. S4A, B). Consistently, cellular staining with the lipophilic dye Oil Red O and BODIPY 493/503 demonstrated that circRPPH1 increased intracellular lipid content in HCC cells (Fig. S4C, D). These findings suggest that circRPPH1 may negatively regulate lipid metabolism by downregulating PPARα in HCC cells. To validate whether circRPPH1 promoted HCC progression by downregulating PPARα, we treated HCC cells overexpressing circRPPH1 with the PPARα agonist pemafibrate. Functionally, CCK8, colony formation, wound healing and Transwell assays revealed that pemafibrate treatment abrogated the promoting effects of circRPPH1 overexpression on HCC cell growth and migration (Fig. 4D-G). Therefore, our data demonstrated that circRPPH1 accelerates HCC cell growth and migration by inhibiting PPARα expression.

### CircRPPH1 inhibits PPARα expression by regulating miR-7845-5p/Sox4 in HCC cells

**Fig. 5:**
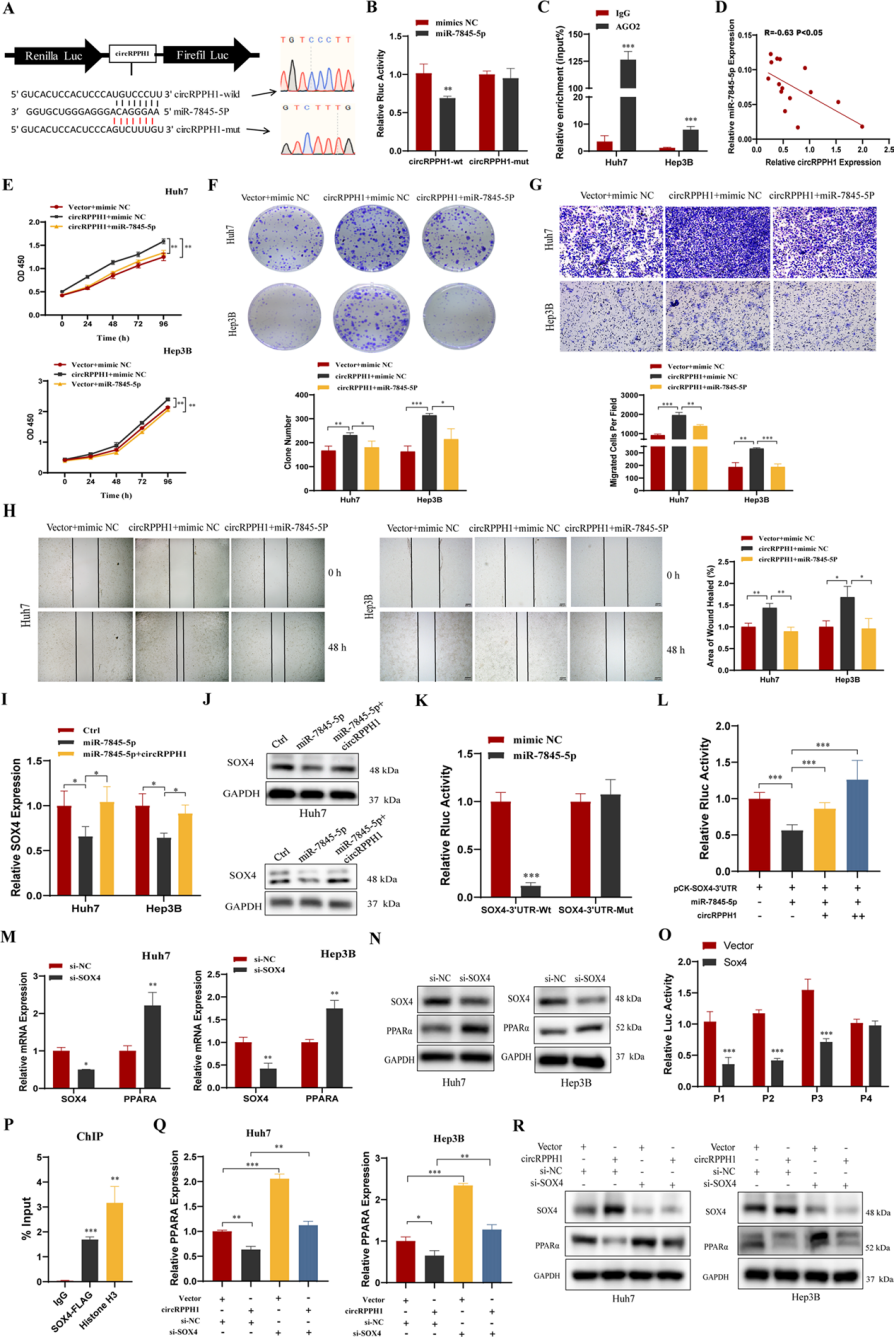
CircRPPH1 upregulates PPARα expression by regulating miR-7845-5p/SOX4 axis. **A** Schematic drawing showed putative binding sites or mutated sites of miR-7845-5p with respect to circRPPH1. **B** Dual luciferase reporter assays in HCC cells co-transfected with wild-type or mutant circRPPH1 reporter and miR-7845-5p mimics. **C** RIP assay to detect the binding of circRPPH1 and AGO2 in Huh7 and Hep3B. **D** Correlation analysis between the expression of circRPPH1 and miR-7845-5p in paired samples of HCC. **E**, **F** Proliferation ability was detected by CCK8 and colony formation assay in HCC cells after co-transfection with circRPPH1 overexpression vector and miR-7845-5p mimics. **G**, **H** Migration ability was detected by transwell assay (scale bar, 100 µm) and wound healing assay (scale bar, 200 µm) in HCC cells after co-transfection with circRPPH1 overexpression vector and miR-7845-5p mimics. **I**, **J** The expression of SOX4 in HCC cells after co-transfection with miR-7845-5p mimics and circRPPH1 overexpression vector using qRT-PCR and Western blot. **K** Dual luciferase reporter assays in HCC cells after co-transfection with wild-type or mutant SOX4 3’ UTR reporter and miR-7845-5p mimics. **L** Detection of luciferase activities of SOX4 3’ UTR in HCC cells after co-transfection with miR-7845-5p mimics and circRPPH1 overexpression vector. **M**, **N** The expression of PPARα in HCC cells after transfection with si-SOX4 using qRT-PCR and Western blot. **O** Luciferase activities detection of PPARα promoter fragments in HCC cells after transfection with sox4 overexpression vector. **P** ChIP-qPCR analysis of SOX4 binding to the PPARα promoter. **Q**, **R** The expression of PPARα in HCC cells after co-transfection with circRPPH1 overexpression vector and siSOX4 using qRT-PCR and Western blot. *P < 0.05, **P < 0.01 and ***P < 0.001

Based on the circRNA-miRNA-mRNA network we constructed in HCC cells (Fig. 1B), we examined whether circRPPH1 functions as a miR-7845-5p sponge to modulate gene expression. The potential binding site between circRPPH1 and miR-7845-5p was visualized using RNAhybrid (Fig. S5A), and corresponding wild-type (WT) and mutant (MUT) luciferase reporter vectors of circRPPH1 were constructed (Fig. 5A). A notable suppression of luciferase activity was observed upon co-transfection of the miR-7845-5p mimic and WT-circRPPH1, whereas no such effect was detected with MUT-circRPPH1 (Fig. 5B). Ago2-mediated RIP assays further confirmed that circRPPH1 acts as a miRNA sponge (Fig. 5C and Fig. S5B). Additionally, we observed that miR-7845-5p expression was downregulated in HCC tissues compared to normal tissues (Fig. S5C, D), and a negative correlation was identified between the expression of circRPPH1 and miR-7845-5p (Fig. 5D). These data confirm that circRPPH1 sponges miR-7845-5p in HCC.

Next, we investigated the functional roles of miR-7845-5p in HCC progression. The results showed that miR-7845-5p mimics significantly inhibited the growth and migration of HCC cells (Fig. S5E-H). Furthermore, we found that the promoting effect of circRPPH1 on the proliferation and migration of HCC cells was rescued by co-transfection with the miR-7845-5p mimic (Fig. 5E-H), suggesting that the oncogenic effects of circRPPH1 in HCC are partially mediated by its function as a sponge for miR-7845-5p.

To identify the target genes of miR-7845-5p, we screened the genes within the circRPPH1-miR-7845-5p-mRNA network, including GREM2, KIFC1, and SOX4. The mRNA and protein levels of SOX4 were decreased in the miR-7845-5p mimic group, and circRPPH1 overexpression reversed the suppressive effect of miR-7845-5p on SOX4 (Fig. 5I, J). However, no such effects were observed for GREM2 and KIFC1 (Fig. S5I). Furthermore, luciferase reporter assays confirmed that SOX4 is a target of miR-7845-5p (Fig. S5J). Subsequently, we constructed WT and MUT luciferase reporter vectors for SOX4 based on the binding sites between miR-7845-5p and SOX4 (Fig. S5K), and the results verified the interaction between SOX4 and miR-7845-5p (Fig. 5K). Importantly, SOX4 luciferase activity was significantly restored by circRPPH1 in a dose-dependent manner upon co-transfection with the miR-7845-5p mimic (Fig. 5L). SOX4 has been recognized as an oncogene [22]. Consistently, analysis of the TCGA database revealed that SOX4 expression was markedly elevated in HCC samples, and patients with higher SOX4 expression exhibited poorer overall survival (Fig. S5L, M). Furthermore, a negative correlation was observed between SOX4 and miR-7845-5p expression (Fig. S5N). All these findings demonstrated that SOX4 is a downstream target of the circRPPH1/miR-7845-5p axis.

Given that SOX4 is an essential transcription factor regulating multiple biological processes, we further investigated the regulatory role of the circRPPH1/miR-7845-5p/SOX4 axis in PPARα. We found that both mRNA and protein levels of PPARα were increased in the miR-7845-5p mimic group, whereas circRPPH1 overexpression reversed this effect (Fig. S5O, S5P). Additionally, knockdown of SOX4 increased the expression of PPARα (Fig. 5M, N). To elucidate the mechanism by which SOX4 regulates PPARα, we first predicted potential SOX4 binding sites in the PPARα promoter region using JASPAR database (Fig. S5Q). A series of truncated fragments of the PPARα promoter were then cloned into the pGL3-Basic vector to assess promoter activity (Fig. S5R). Luciferase reporter assay revealed that SOX4 significantly inhibited PPARα promoter activity, with the primary inhibitory region mapped to the − 540 bp to -1500 bp segment (Fig. 5O). Consistently, ChIP-qPCR assays demonstrated that SOX4 could bound to the PPARα gene promoter in HCC cells, confirming direct transcriptional repression (Fig. 5P). Moreover, knockdown of SOX4 reversed the decrease in PPARα level induced by circRPPH1 overexpression (Fig. 5Q, R). Collectively, these results indicate that circRPPH1 inhibits PPARα expression by regulating the miR-7845-5p/SOX4 axis in HCC cells.

### CircRPPH1 interacts with USP1 to ubiquitinate and destabilize PPARα

**Fig. 6:**
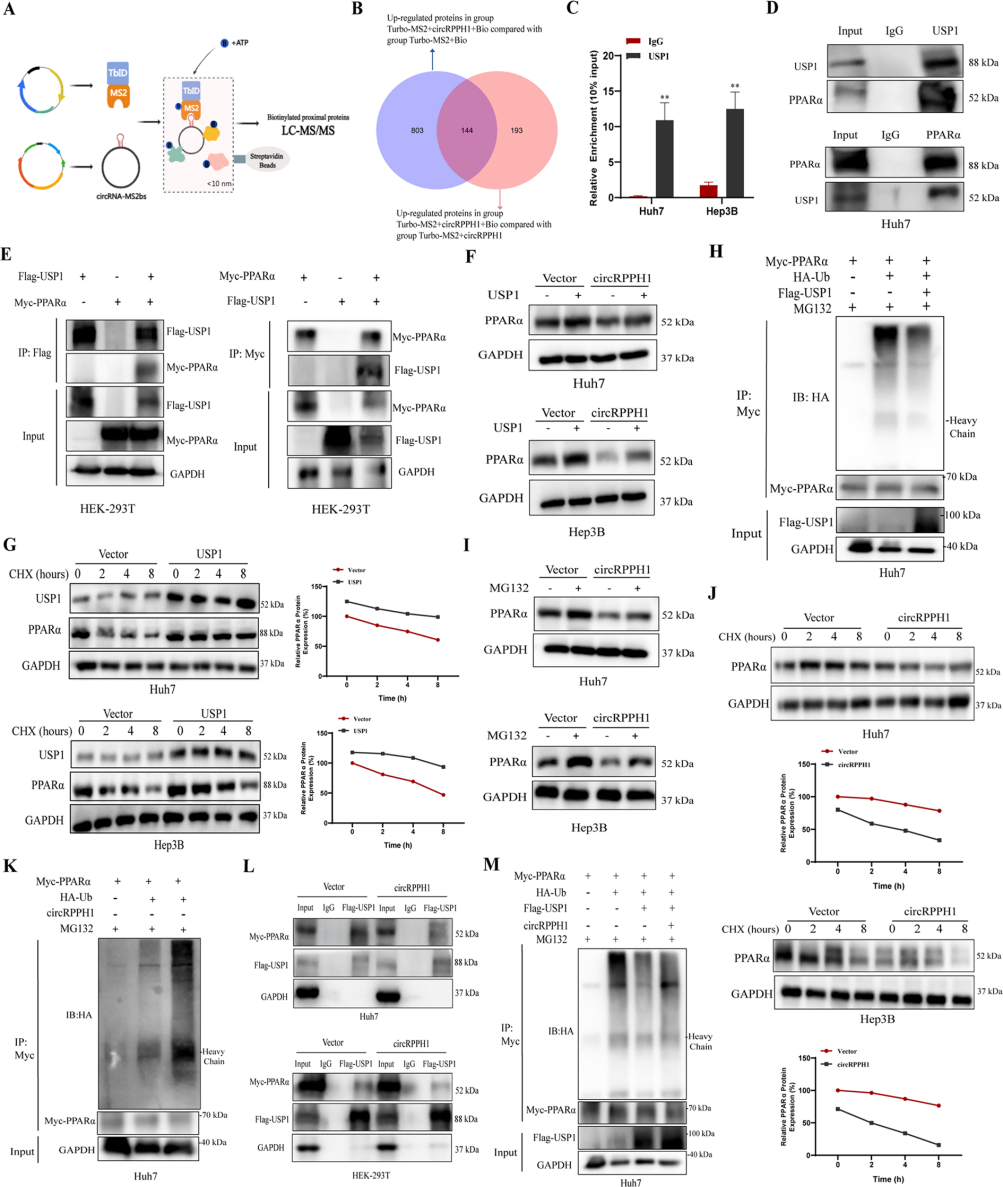
CircRPPH1 competitively bound with USP1 to ubiquitinate and destabilize PPARα. **A** Schematic drawing showed circRNA-TurboID proximity labeling system. **B** Determine the number of proteins interacting with circRPPH1 using Venn diagram. **C** RIP assay to detect the binding of circRPPH1 and USP1 in Huh7 and Hep3B. **D** USP1 was immunoprecipitated from Huh7 cells and immunoblotted to PPARα. **E** Flag-tagged or Myc-tagged protein was immunoprecipitated from HEK293T cells after transfection with plasmids encoding either Flag-USP1 or Myc-PPARα, alone or in combination, and then immunoblotted with Myc or Flag antibodies. **F** The protein level of PPARα in Huh7 cells after co-transfection with USP1 and circRPPH1 overexpression vector using Western blot. **G** Detection of PPARα protein levels in HCC cells transfected with USP1 overexpression vector following CHX (40 µM) treatment for the indicated times. **H** Huh7 cells were co-transfected with Myc-PPARα, HA-Ub, Flag-USP1 and cell lysates were subjected to IP with Myc beads followed by IB analysis with antibodies against HA and Myc. Cells were treated with MG132 (10 μm) for 8 h before harvesting. **I**, **J** Detection of PPARα protein level after treatment with MG132 (10 µM) or CHX (40 µM) in HCC cells transfected with circRPPH1 overexpression vector. **K** Huh7 cells were co-transfected with Myc-PPARα, HA-Ub, circRPPH1 overexpression vector, and cell lysates were subjected to IP with Myc beads followed by IB analysis with antibodies against HA and Myc. Cells were treated with proteasome inhibitor MG132 (10 μm) for 8 h before harvesting. **L** Detection of interaction between PPARα and USP1 in Huh7 cells co-transfected with Myc-PPARα, Flag-USP1 and circRPPH1 overexpression vector through IP with Flag beads. **M** Huh7 cells were co-transfected with Myc-PPARα, HA-Ub, Flag-USP1, circRPPH1 overexpression vector, and cell lysates were subjected to IP with Myc beads followed by IB analysis with antibodies against HA and Myc. Cells were treated with MG132 (10 μm) for 8 h before harvesting. **P < 0.01

Accumulating evidence has demonstrated that circRNAs can also function as protein decoys, thereby modulating protein-protein interactions [23]. To identify potential proteins that bind to circRPPH1, we developed the circRNA-TurboID proximity labeling system, and biotin-labeled proteins in close proximity to circRPPH1 were analyzed by LC-MS/MS (Fig. 6A and Fig. S6A). We identified 144 unique proteins that were specifically labeled by circRPPH1, but not by the control group (Fig. 6B). Gene Ontology (GO) enrichment analysis revealed that these proteins were predominantly enriched in metabolic processes (Fig. S6B). Interestingly, the deubiquitinase USP1 was identified in the prey list (Fig. S6C). Western blot analysis was further performed to validate the mass spectrometry results (Fig. S6D). HDOCK was utilized for the docking simulation of the three-dimensional structures of circRPPH1 and USP1 (Fig. S6E). Subsequently, the specific interaction between circRPPH1 and USP1 was confirmed by RIP assays (Fig. 6C). Furthermore, we found that circRPPH1 overexpression did not affect USP1 expression (Fig. S6F, G).

A recent study has revealed that USP25 directly interacts with and deubiquitinates PPARα to enhance PPARα stability in metabolic dysfunction-associated steatotic liver disease (MASLD) [24].Consequently, we focused on investigating whether PPARα is a target of USP1 deubiquitinase activity in HCC. The specific interaction between USP1 and PPARα was confirmed through co-immunoprecipitation (Co-IP) assays in Huh7 cells, as well as in HEK293T cells with exogenous expression of Flag-tagged USP1 and Myc-tagged PPARα (Fig. 6D, E and Fig. S6H). To investigate whether USP1 regulates PPARα protein stability, we first overexpressed USP1 in HCC cells and observed a corresponding increase in PPARα protein levels (Fig. 6F). Next, cycloheximide (CHX) chase assays revealed that USP1 overexpression extended the half-life of PPARα (Fig. 6G). Furthermore, we detected the effect of USP1 on PPARα ubiquitination in Huh7 cells and observed that USP1 significantly promoted PPARα deubiquitination (Fig. 6H).

Notably, overexpression of circRPPH1 counteracted the USP1-mediated upregulation of PPARα expression (Fig. 6F). Therefore, we hypothesize that circRPPH1 may promote PPARα degradation through a ubiquitination-dependent pathway by disrupting the interaction between USP1 and PPARα. In HCC cells, treatment with the proteasome inhibitor MG132 partially reversed the decrease in PPARα protein levels induced by circRPPH1 overexpression (Fig. 6I). Moreover, circRPPH1 overexpression markedly decreased the stability of endogenous PPARα protein in the presence of CHX (Fig. 6J). Ubiquitination assays further revealed that PPARα ubiquitination was enhanced by circRPPH1 overexpression (Fig. 6K). To elucidate the underlying mechanism, we carried out Co-IP assays, and the results demonstrated that circRPPH1 overexpression weakened the interaction between USP1 and PPARα in Huh7 and HEK293T cells (Fig. 6L). Notably, the decrease in PPARα ubiquitination induced by USP1 overexpression was reversed by enforced circRPPH1 expression (Fig. 6M). Collectively, these results suggest that circRPPH1 promotes PPARα ubiquitination and degradation by disrupting the interaction between USP1 and PPARα in HCC cells.

### CircRPPH1 promotes the HCC growth in vivo

**Fig. 7:**
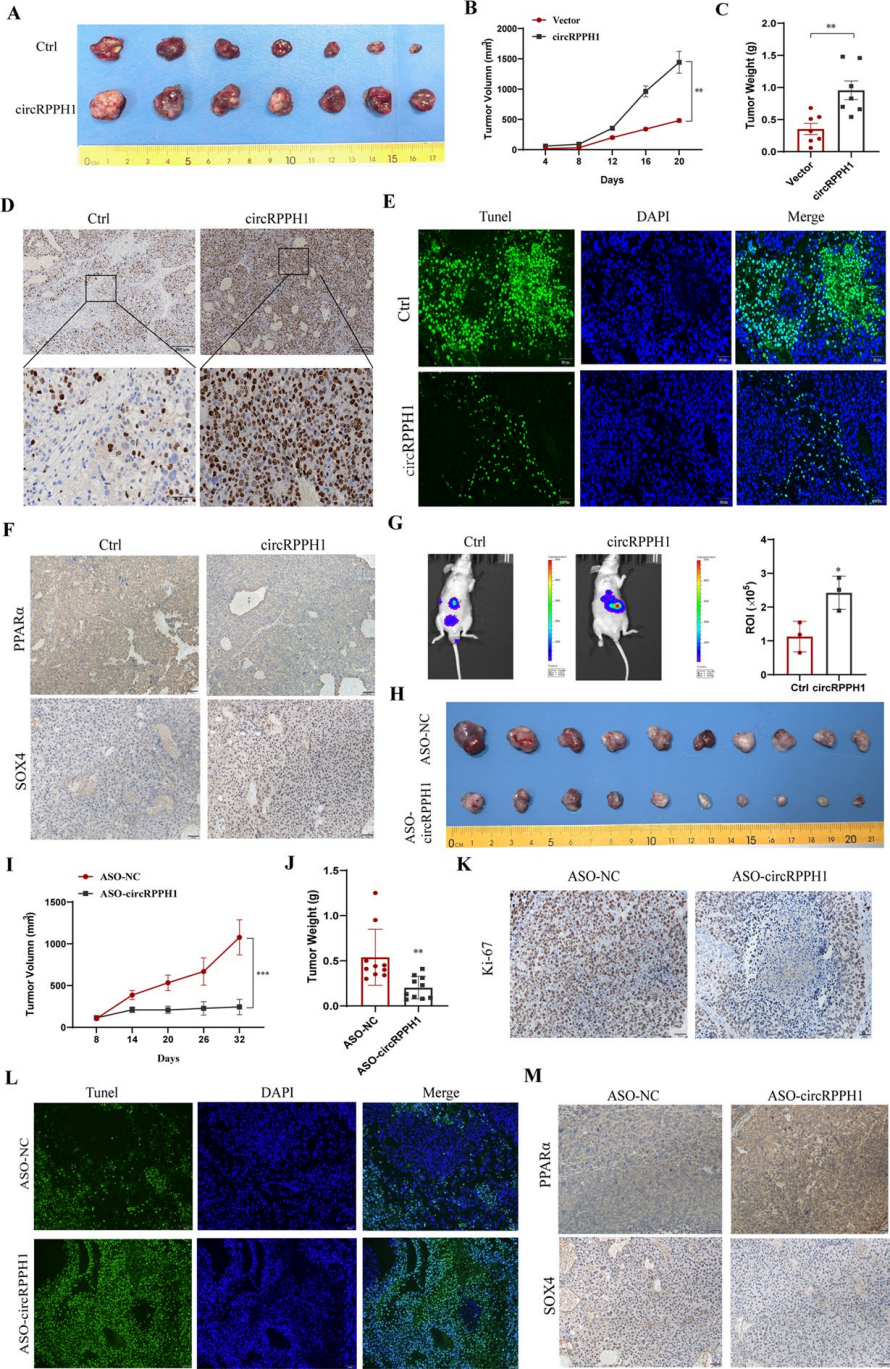
CircRRPPH1 promotes HCC growth, whereas circRRPPH1-ASO suppresses HCC growth *in vivo*. **A** Representative images of subcutaneous xenograft tumors. **B**, **C** Comparison of tumor volume and weight between circRRPPH1 overexpression and NC groups. **D**, **E** Ki-67 and TUNEL staining of subcutaneous xenograft tumors (scale bar, 50 μm) in the tumor tissues from circRRPPH1 overexpression and NC groups. **F** Immunohistochemistry (IHC) staining of PPARα and SOX4 in the tumor tissues from circRRPPH1 overexpression and NC groups. **G** Representative bioluminescence imaging (left) and standardized region of interest (ROI) to show the growth of orthotopic HCC tumor in circRRPPH1 overexpression and NC groups. **H** Representative images of subcutaneous xenograft tumors in ASO-circRRPPH1 and ASO-NC groups. **I**, **J** Comparison of tumor volume and weight between ASO-circRRPPH1 and ASO-NC groups. **K**, **L** Ki-67 and TUNEL staining of subcutaneous xenograft tumors (scale bar, 50 μm) in the tumor tissues from ASO-circRRPPH1 and ASO-NC groups. **M** Immunohistochemistry staining of PPARα and SOX4 in the tumor tissues from ASO-circRRPPH1 and ASO-NC groups. *P < 0.05, **P < 0.01 and ***P < 0.001

To investigate the role of circRPPH1 in HCC growth in vivo, we established subcutaneous xenograft models using Huh7 cells stably overexpressing circRPPH1. Monitoring tumor growth revealed that circRPPH1 overexpression significantly accelerated tumor growth and increased tumor weights compared with the control group (Fig. 7A-C). Ki-67 and TUNEL staining further revealed that the tumors derived from cells overexpressing circRPPH1 exhibited increased proliferation and decreased apoptosis (Fig. 7D, E). Immunohistochemical (IHC) analysis revealed decreased PPARα expression and increased SOX4 expression in the circRPPH1-overexpressing group (Fig. 7F). Additionally, decreased CPT1A expression and increased FASN expression were observed, suggesting that circRPPH1 suppresses lipid metabolism (Fig. S7A). To further validate these findings, we established an orthotopic HCC tumor model by injecting Huh7 cells overexpressing circRPPH1 into the liver. In vivo imaging showed that orthotopic tumors derived from circRPPH1-overexpressing cells grew significantly faster than those in the control group, as evidenced by enhanced fluorescence signals (Fig. 7G).

To further validate the therapeutic potential of circRPPH1 in HCC, we designed two antisense oligonucleotides (ASOs) to target and degrade circRPPH1. The expression of circRPPH1 was effectively inhibited by circRPPH1-ASO1 in Huh7 cells (Fig. S7B). Next, we established a subcutaneous xenograft model to confirm the therapeutic efficacy of circRPPH1-ASO1 in vivo (Fig. S7C). As expected, the results indicated that mice treated with circRPPH1-ASO1 exhibited significantly decreased tumor growth rates and tumor weights compared with the ASO-control group (Fig. 7H-J). Histological analyses revealed a reduction in the number of proliferative cells and an increase in the number of apoptotic cells in xenograft tumors treated with circRPPH1-ASO1 (Fig. 7K, L). Moreover, PPARα expression was increased, and SOX4 expression was decreased in tumors from the circRPPH1-ASO1 group (Fig. 7M). Together, our data demonstrate that circRPPH1 promotes HCC growth, and may serve as a potential therapeutic target for HCC treatment.

## Discussion

There is accumulating evidence that dysregulated expression of circRNAs plays a pivotal role in the development and progression of various cancers, including HCC, and that these molecules hold promise as diagnostic biomarkers and therapeutic targets in cancer. To further investigate the roles of circRNAs in HCC progression, we constructed a circRNA-miRNA-mRNA network and screened for 6 co-expressed circRNAs, 6 miRNAs, and 52 mRNAs. Among these candidate circRNAs, we first identified circRPPH1 (hsa_circ_0000520) as being significantly upregulated in HCC tissues. Functional experiments revealed the promoting effect of circRPPH1 in HCC progression both in vitro and in vivo. Previous studies have reported that diverse circRNAs deriving from distinct regions of RPPH1 gene can promote tumor progression. For instance, circRPPH1 (hsa_circRNA_000166) has been shown to promote the progression of triple-negative breast cancer (TNBC) by acting as a sponge for miR-326, thereby activating ITGA5-induced FAK/PI3K/AKT pathway [17]. CircRPPH1 (hsa_circ_0000512) facilitates the malignant phenotype and stemness of glioma stem cells (GSCs) through the UPF1/circRPPH1/ATF3 feedback loop [18]. CircRPPH1 (hsa_circ_0000515) promotes lung adenocarcinoma (LUAD) progression by binding to MAFK and modulating SIRT gene-mediated cellular pyroptosis [19]. Our findings, which emphasized the oncogenic role of circRPPH1 in HCC progression, are consistent with these studies and suggest that circRPPH1 may serve as a potential therapeutic target for cancer. Notably, while RPPH1 mRNA shows no significant difference between HCC and normal tissues, circRPPH1 is markedly upregulated and acts as an oncogenic factor in HCC, highlighting the important characteristic of circRNAs functioning independently of their parental gene.

Beyond its role in regulating mRNA, m^6^A modification occupies a pivotal position in sustaining the biological activity of circRNA [10]. Alterations in m^6^A levels impact circRNA biosynthesis. In non-small-cell lung cancer (NSCLC), METTL3/YTHDC1 regulate m^6^A-mediated circIGF2BP3 biogenesis by promoting its back-splicing [9]. In HCC, circCPSF6 is regulated by ALKBH5-mediated demethylation, thereby enhancing its RNA stability [25]. Consequently, m^6^A modification functions as a key molecular regulator of circRNA biogenesis. In exploring the upstream regulation mechanism of circRPPH1, we identified an m^6^A site within circRPPH1. Further study confirmed that m^6^A modification influences the expression of circRPPH1 and is dependent on METTL3. Furthermore, m6A-modified circRPPH1 binds to YTHDC1, which stabilizes circRPPH1 and enhances its expression in HCC cells. This finding elucidates the mechanism underlying the upregulation of circRPPH1 in HCC.

Recently, abnormalities in the PPAR signaling pathway have been increasingly recognized as a mechanism involved in HCC initiation and progression [26]. In our RNA-seq analysis, we observed that the PPAR signaling changed most significantly, whereas the fat digestion and cholesterol metabolism pathways were also significantly enriched. Accordingly, to verify the mechanism by which circRPPH1 regulates HCC progression, we examined the effects of circRPPH1 on PPAR isotypes. PPARα is predominantly highly expressed in the liver, and plays a crucial role in coordinating the metabolism of fatty acids and glucose [2]. Notably, dysregulation of PPARα in HCC results in abnormal FAO metabolism and aggravation of hepatic steatosis [11]. Suppression of FAO promotes the growth of HCC cells, consistent with reported downregulation of PPARα expression in HCC samples [12, 27]. Previous studies have demonstrated that PPARα exhibits tumor-suppressive properties in HCC [12, 14, 15, 28]. Consistently, we found that circRPPH1 significantly downregulated expression of PPARα, as well as its transcriptional targets, leading to lipid accumulation and HCC development. Moreover, treatment with a PPARα agonist counteracted the proliferation and migration induced by circRPPH1 overexpression in HCC cells, suggesting that circRPPH1 exerts a tumor-promoting role by suppressing PPARα-mediated lipid metabolism.

However, it has been reported that PPARα promotes tumorigenesis in HCC [29–31]. PPARα exhibits context-dependent dual roles in HCC progression, which may be determined by the metabolic state, dominant signaling pathways, and regulatory factor. Although some studies have suggested that PPARα agonists, such as fenofibrate, may promote HCC progression, other paradoxical studies have demonstrated that fenofibrate may exhibit more pronounced anti-tumor effects in the liver [32–34]. Proliferating cancer cells have substantial lipid requirements for cellular membrane synthesis and other essential functions [11, 12, 35]. Intratumoral immune cells also exhibit increased lipid absorption and accumulation, which is frequently associated with impaired anti-tumor immune responses [36, 37]. Building upon the established link between PPARα deficiency and hepatic lipid dysregulation, our findings further demonstrated that PPARα promotes HCC progression through suppression of lipid metabolism.

With the emergence of circRNAs as potent regulatory factors, numerous studies have confirmed a close correlation between competing endogenous RNAs (ceRNA) crosstalk and tumorigenesis in HCC. For instance, circPIAS1 inhibited ferroptosis by competitively binding to miR-455-3p, thereby exacerbating the progression of HCC [8]. We constructed and visualized the circRNA-miRNA-mRNA network associated with HCC, which facilitated the identification of potential circRNA-related ceRNA crosstalk in HCC. The circRPPH1-miR-7845-5p-SOX4 axis was conclusively identified as a regulatory mechanism in HCC. We have demonstrated, for the first time, that miR-7845-5p effectively inhibits the proliferation and migration of HCC cells. Furthermore, cotransfection with miR-7845-5p partially reverses the stimulatory effect of circRPPH1 on HCC progression, suggesting a mechanism whereby circRPPH1 sequesters miR-7845-5p in HCC. SOX4 is consistently overexpressed in multiple cancer types and has been functionally validated as an oncogene [22]. Previous studies have reported that SOX4 is directly targeted by several tumor-suppressive miRNAs, including miR-19a-3p, miR-449, miR-138-5p, miR-363-3p, and miR-130a-3p, which collectively regulate its oncogenic activity in HCC [38–42]. Our results demonstrated that SOX4 is a potential target of miR-7845-5p in HCC, and circRPPH1 enhances the expression of SOX4 by adsorbing miR-7845-5p. Based on SOX4’s transcriptional regulatory function, we further investigated its regulatory impact on PPARα. As expected, we found that SOX4 effectively suppresses the transcription of PPARα. Furthermore, the circRPPH1-SOX4-PPARα regulatory axis was validated in vivo, where SOX4 expression was positively correlated with circRPPH1, and PPARα expression was negatively correlated with circRPPH1. This study conclusively indicates the role of circRPPH1 as a miR-7845-5p sponge to increase SOX4 expression, which in turn inhibited PPARα transcription in HCC cells.

Additionally, accumulating evidence suggests that circRNAs can also function as protein scaffolds during cancer progression. CircMPP6 acted as a scaffold to facilitate the interaction between MEX3A and three PBs-related proteins, promoting PDE5A mRNA degradation and the aggressive properties of colorectal cancer (CRC) [43]. CircWSB1 can bind to USP10 and disrupt the interplay between USP10 and p53, leading to the poly-ubiquitination and subsequent degradation of p53 in NSCLC cells [44]. Analogous to this study, we have discovered that circRPPH1 competitively binds to USP1 without affecting its expression level, thereby disrupting the interaction between USP1 and PPARα, which subsequently enhances the ubiquitination and degradation of PPARα. USP1 is a de-ubiquitinase that functions as a regulator of DNA damage response, tumorigenesis, and immune response [45]. Interestingly, while previous studies have identified USP1 deubiquitination targets as pro-oncogenic, our findings demonstrated that in the context of overexpressed circRPPH1, the failure of USP1 to protect PPARα results in an alternative oncogenic mechanism [46, 47]. This indirect tumor-promoting effect observed for USP1 in our findings underscores its context-dependent functionality and suggests the regulatory diversity and complexity of deubiquitination in the development of various cancers.

Given the oncogenic role of circRPPH1 in HCC, it may serve as a potential therapeutic target, providing new avenues for treating HCC. RNA interference (RNAi)-based therapeutics have emerged as a groundbreaking class of drugs, holding considerable promise in oncology therapeutic domains [48]. In particular, the development of ASO targeting circRNA represents a promising strategy for cancer therapy [49, 50]. Our results indicated that circRPPH1-ASO effectively reduced HCC growth in vivo.

There are limitations in our study. Multi-omics analyses, including the construction of circRNA-miRNA-mRNA network, transcriptomic profiling following circRPPH1 overexpression, and mass spectrometry of circRPPH1-interacting proteins, consistently demonstrated the significant association between circRPPH1 and metabolic pathways, with particular emphasis on lipid metabolism. Based on these findings, we focused our investigation on circRPPH1-mediated regulation of PPARα signaling. However, three major fatty acid oxidative metabolic pathways, including mitochondrial β-oxidation, peroxisomal β-oxidation, and microsomal ω-oxidation are regulated by PPARα [2]. The specific downstream lipid metabolic pathways regulated by the circRPPH1-PPARα axis remain to be fully elucidated.

## Conclusion

**Fig. 8:**
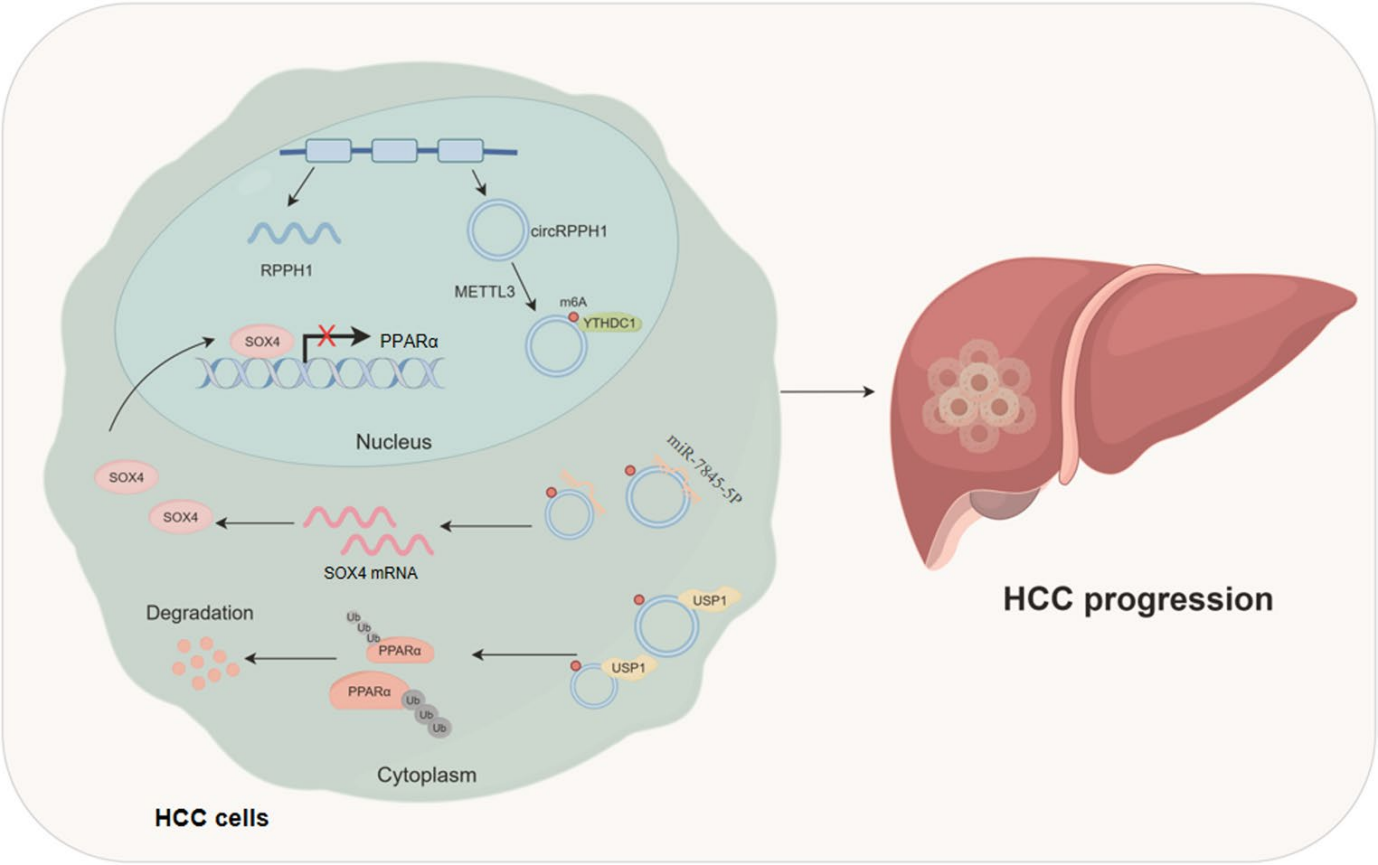
Schematic diagram illustrating the mechanism by which circRPPH1 promotes HCC progression

In summary, we have revealed that m^6^A-modified circRPPH1 functions as a cancer-promoting factor in HCC. Furthermore, circRPPH1 inhibits PPARα transcription via the miR-7845-5p/SOX4 axis and accelerates the degradation of PPARα by inhibiting USP1-mediated PPARα stabilization (Fig. 8). Our findings facilitates a better understanding of HCC tumorigenesis from the perspective of circRNAs.

## Supplementary Information

Below is the link to the electronic supplementary material.

Supplementary Material 1

## Data Availability

The authors declare that data supporting the findings of this study are available within the article and its supplementary information files.

## Abbreviations

GEO: Gene Expression Omnibus
AGO2: Argonaute 2
DAPI: 4’,6-diamidino-2-phenylindole
FISH: Fluorescence in Situ Hybridization
IHC: Immunohistochemistry
KEGG: Kyoto Encyclopedia of Genes Genomes
miRNAs: MicroRNAs
MS: Mass Spectrometry
ncRNAs: Non-coding RNAs
TCGA: The Cancer Genome Atlas
CHX: cycloheximide
RIP: RNA immunoprecipitation
Co-IP: Co-immunoprecipitation
ASO: Antisense oligonucleotide
TUNEL: terminal deoxynucleotidyl transferase-mediated dUTP-biotin nick end labeling
