# Supplementary material for: CircRPPH1 promotes hepatocellular carcinoma progression through inhibiting PPARα transcription and accelerating its ubiquitination and degradation
Source: Cell Mol Life Sci. 2026 Apr 30;83(1):261. doi: 10.1007/s00018-026-06211-3 (PMC13287188; doi:10.1007/s00018-026-06211-3)
Supplement: Supplementary file 1 — Supplementary Material 1 [file 18_2026_6211_MOESM1_ESM.doc]

**CircRPPH1 promotes hepatocellular carcinoma** **progression through inhibiting PPARα transcription and accelerating its ubiquitination and degradation**

Wenxiu Ru, Yu Lu, Lunbiao Gan, Siyu Yao, Huanhuan Wan, Fengze Nie, Sijia Di, Yujing Guo, Qian Huang, Sha Yin, Jiaqi Ning, Fa Yang, Weijun Qin, Weihong Wen

Supplementary methods……………………………………………………..……………2

Supplementary figures………………………………………………………….………...4

Supplementary tables……………………………………………...……………………..11

Supplementary reference……………………………………………………………...…12

**Supplementary methods**

**Reverse transcription and qRT-PCR**

Total RNA was extracted using TRIzol reagent (AG, China) and cDNAs were generated using RT reagent kit (AG, China) according to the manufacturer’s protocol. qPCR was conducted on a Real-time PCR Detection System (Bio-Rad, USA) with SYBR Green mix (YEASEN, China). Relative mRNA, circRNA and miRNA expression were normalized to GAPDH or U6. Each sample was replicated three times and quantitation data was calculated by the 2^-ΔΔCt^ method. Primer sequences are provided in the supplementary Table [S1](https://molecular-cancer.biomedcentral.com/articles/10.1186/s12943-024-02038-3#MOESM1).

**RNase R and Actinomycin D treatment**

Total RNA was digested with RNase R (Beyotime, China) at 37°C for 3 h. Actinomycin D (Sigma, Germany) at 2 μg/ml was added to the medium of cells to measure RNA stability. The treated RNA was reverse-transcribed into cDNA, which was then analyzed by qRT-PCR assay

**CCK-8 assay, EdU assay, colony formation assay**

The growth curves of HCC cells were evaluated using CCK-8 kit (YEASEN, China) according to the protocols of manufacturer. For colony formation, the transfected HCC cells (1,000/well) were cultured in 6-well plates for two weeks. The colonies were fixed with 4% paraformaldehyde and stained with 0.5% crystal violet (Beyotime, China). Cell proliferation was also evaluated by EdU assay kit (Beyotime, China) according to the manufacturer’s instructions and the images were obtained by fluorescence microscopy (OLYMPUS, Japan). All experiments were performed at least in triplicate.

**Transwell assay and wound healing assay**

The transfected cells (50,000 cells/well) were resuspended in 200 µL of serum-free medium and seeded into the upper chamber of 24-well Transwell plates with 8 μm-pore size (Corning, USA), while the lower chamber was filled with 600 µL of complete medium. After 48 h, the cells on the upper surface were removed by scrubbing, and migrating cells on the bottom surface were fixed with 4% paraformaldehyde and 0.5% crystal violet (Beyotime, China). For wound healing assay, the transfected cells were were cultured in 6-well plates to full confluency. Subsequently, cells were scratched, and then cultured in serum-free medium for 48 hours. Images were taken at 0 h and 48 h document wound width changes. All experiments were performed at least in triplicate.

**Western blot**

The cells were lysed with the protein extraction reagent RIPA containing 1 mM PMSF (Beyotime, China), and the protein concentration was quantified using the BCA protein assay kit (Beyotime, China). The extracted proteins were separated by 10% SDS-PAGE gels and transferred to 0.45 μm PVDF membranes (Millipore, Germany). After blocking with 5% non-fat milk, the membranes were incubated overnight at 4°C with primary antibodies, followed by their HRP-labeled secondary counterparts. The immunoreactive bands were detected using a chemiluminescence system (Vazyme, China).

**Dual-luciferase reporter assay**

Reporter plasmids containing the wild-type or mutant binding site for miR-7845-5p were generated by inserting sequences from circRPPH1 and 3’UTR of SOX4 into the psi-Check2 vector. Additionally, the PPARα promoter sequence was inserted into the pGL3-reporter vector. These reporter vectors were then transfected into HEK293T cells, and luciferase activities of all reporter vectors were detected using Dual-Luciferase Reporter Assay Kit (UE, China) according to the manufacturer’s instructions. All experiments were performed at least in triplicate.

**Co-immunoprecipitation assay (Co-IP)**

The transfected cells were lysed with IP lysis (Beyotime, China) supplemented with PMSF (Beyotime, China) for 20 min on ice, then centrifugated at 14,000 g for 20min to collect supernatant. The supernatant were incubated with specific antibodies and anti-IgG for 4 h at 4°C. Subsequently, the lysate/antibody mix was incubated overnight at 4°C with protein A/G magnetic beads (Thermo, Waltham). After washing, the co-immunoprecipitated proteins were eluted with SDS-PAGE sample loading buffer (Beyotime, China) and heated at 100°C for 10 min, then followed by western blot.

**Immunohistochemistry**

An established protocol as described in previous studies was followed in this study [1]. Briefly, tissues were fixed in formalin, dehydrated in ethanol, embedded with paraffin. Slides were incubated with primary antibodies against Ki-67, PPARα, and SOX4 at 4°C overnight followed by incubation with the secondary antibody at room temperature. Then, DAB and hematoxylin staining were carried out, followed by photographing under a microscope.

**Supplementary figures**

**Figure S1 (related to Figure 1).** (A) KEGG analysis of differentially expressed genes in circRNA-miRNA-mRNA network. (B) Heatmap of top 10 upregulated and top 10 downregulated circRNA in HCC.

**Figure S2** **(related to Figure 2).** (A) The expression of RPPH1 in paired HCC tissues was analysed using TCGA data. (B) The potential m^6^A site on circRPPH1 was predicted by SRAMP. (C) The relative expression of METTL3 and METTL14 in HCC cells after treatment with siMETTL3 or siMETTL14 using qRT-PCR. (D) The expression of circRPPH1 in siMETTL14 HCC cells using qRT-PCR. (E) The expression of METTL3 in paired HCC and NATs using qRT-PCR. (F, G) The expression of circRPPH1 in siIGF2BP1 and siIGF2BP3 HCC cells using qRT-PCR. (H) The expression of YTHDC1 in paired HCC and NATs using qRT-PCR. Data were represented the mean±SD. **P*< 0.05, ***P*< 0.01 and ****P*< 0.001 were determined by Student’s t test.

**Figure S3** **(related to Figure 3).** (A) The efficiency of circRPPH1 overexpression in HepG2 and Huh7 cells. (B) The efficiency of circRPPH1 knockdown in MHCC-LM3 and Hep3B cells. Data were represented the mean±SD. **P*< 0.05, ***P*< 0.01 and ****P*< 0.001 were determined by Student’s t test.

**Figure S4** **(related to Figure 4).** (A, B) qRT-PCR analysis for expression of lipid metabolism enzymes and lipogenic enzyme genes in HCC cells overexpressing circRPPH1. (C) Representative images of oil red o and BODIPY staining in HCC cells overexpressing circRPPH1. Scale bars, 50 μm. Data were represented the mean±SD. **P*< 0.05, ***P*< 0.01 and ****P*< 0.001 were determined by Student’s t test.

**Figure S5** **(related to Figure 5).** (A) The proposed circRPPH1 binding site on miR-7845-5p was identified using RNAhybrid. (B) RIP assay to detect the binding of circRPPH1 and AGO2 in HCC cells. (C) The expression of miR-7845-5p in paired HCC tissues was analysed using TCGA data. (D) The expression of miR-7845-5p in paired HCC and NATs using qRT-PCR. (E, F) Proliferation ability of HCC cells was detected by CCK8 and colony formation assay after transfection with miR-7845-5p mimics. (G, H) Migration ability was detected by transwell assay (scale bar, 100 μm) and wound healing assay (scale bar, 200 μm) in HCC cells after transfection with miR-7845-5p mimics. (I) The expression of KIFC1 and GREM2 in HCC cells after co-transfection with miR-7845-5p mimics and circRPPH1 overexpression vector using qRT-PCR. (J) Detection of luciferase activities of SOX4 3' UTR, KIFC1 3' UTR and GREM2 3' UTR in HCC cells after transfection with miR-7845-5p mimics. (K) Putative binding sequences of miR-7845-5p on SOX4 3' UTR. (L) The expression of SOX4 in paired HCC tissues was analysed using TCGA data. (M) Kaplan-Meier analysis of overall survival based on SOX4 expression levels in HCC patients using TCGA data. (N) Correlation analysis showed a negative relationship between the levels of SOX4 and miR-7845-5p using TCGA data. (O, P) The expression of PPARα in HCC cells after co-transfection with miR-7845-5p mimics and circRPPH1 overexpression vector using qRT-PCR and western blot. (Q) Putative binding sequences of SOX4 on PPARα promoter. (R) Schematic Diagram of PPARα promoter fragments. Data were represented the mean±SD. **P*< 0.05, ***P*< 0.01 and ****P*< 0.001 were determined by Student’s t test.

**Figure S6** **(related to Figure 6).** (A) Biotinylated proteins was immunoprecipitated by streptavidin magnetic beads and immunoblotted using streptavidin (SA) antibody. (B) KEGG analysis of circRPPH1-interacting proteins. (C) Presenting the top 5 circRPPH1-interacting proteins by mass spectrometry. (D) Biotinylated proteins was immunoprecipitated by streptavidin magnetic beads and immunoblotted to USP1. (E) Establishment of three-dimensional docking model of circRPPH1 and USP1 using [HDOCK](https://zhida.zhihu.com/search?content_id=191831234&content_type=Article&match_order=1&q=HDOCK&zhida_source=entity). (F, G) The expression of USP1 in HCC cells after overexpression circRPPH1 using qRT-PCR and Western blot. (H) Co-IP assay to detect the interaction between Flag-USP1 and Myc-PPARα in HEK293T cells.

**Figure S7** **(related to Figure 7).** (A) Immunohistochemistry (IHC) staining of CPT1A and FASN in the tumor tissues from circRRPPH1 overexpression and NC groups. (B) The efficiency of circRPPH1 knockdown in Huh7 cells after transfection with ASO-circRPPH1. (C) Schematic image to show the process of ASO-circRPPH1 treatment. Data were represented the mean±SD. ****P*< 0.001 were determined by Student’s t test.

**Supplementary tables**

**Table S1. The sequences of primers**

| Gene | Forward primers | Reverse primers |
| --- | --- | --- |
| GAPDH | CATCTTCTTTTGCGTCGCCA | TTAAAAGCAGCCCTGGTGACC |
| CircRPPH1 | TTGGGAAGGTCTGAGACTAGGG | CCACTGATGAGCTTCCCTCC |
| RPPH1 | CGAGCTGAGTGCGTCCTGTC | TCGCTGGCCGTGAGTCTGT |
| METTL3 | TTGTCTCCAACCTTCCGTAGT | CCAGATCAGAGAGGTGGTGTAG |
| METTL14 | GAACACAGAGCTTAAATCCCCA | TGTCAGCTAAACCTACATCCCTG |
| YTHDC1 | ATCTTCCGTTCGTGCTGTCC | GGACCATACACCCTTCGCTT |
| IGF2BP1 | AGGCAGGCTGACGAGGTTCC | GGTTCCGTCCTTCCTTGCCAATG |
| IGF2BP3 | TCACTTCTATGCTTGCCAGGTTGC | CCTTCTGTTGTTGGTGCTGCTTTAC |
| PPARA | ATGGTGGACACGGAAAGCC | CGATGGATTGCGAAATCTCTTGG |
| PPARD | CAGGGCTGACTGCAAACGA | CTGCCACAATGTCTCGATGTC |
| PPARG | CCCAGAAAGCGATTCCTTCAC | GCAGGCTCCACTTTGATTGC |
| ACACA | ATGTCTGGCTTGCACCTAGTA | CCCCAAAGCGAGTAACAAATTCT |
| ACLY | TCGGCCAAGGCAATTTCAGAG | CGAGCATACTTGAACCGATTCT |
| FASN | AAGGACCTGTCTAGGTTTGATGC | TGGCTTCATAGGTGACTTCCA |
| SCD1 | TCTAGCTCCTATACCACCACCA | TCGTCTCCAACTTATCTCCTCC |
| ACOX1 | TGCTCAGAAAGAGAAATGGC | TGGGTTTCAGGGTCATACG |
| CPT1A | CCTCCGTAGCTGACTCGGTA | CGGAGTGACCGTGAACTGA |
| CPT2 | GCCTAGATGACTTCCCCATTAA | AAAGGATTTATCAAACCAGCGG |
| ECHS1 | GCCTCGGGTGCTAACTTTGA | GCCATCGCAAAGTGCATTGA |
| miR-7845-5p | TGCAAGGGACAGGGAGGG | GTGCAGGGTCCGAGGT |
| SOX4 | GACTTCGAGTTTGCTCCCCT | TAACTCGCCTTCTTGCTGGG |
| GREM2 | TCACGTCATTGCAGGATGTTC | CTTGTAAGGCGAGGGGATGG |
| KIFC1 | CGTGCGAGTTCTCTACCCTG | GACCTCTGCGGATCCATGTC |
| USP1 | TTCGGTTGAACAGCTCCAGG | GGGTTGAGTTCCCTCAGTGT |
| PPARA  (ChIP-qPCR) | CTCCAGTTCCCCTGCCTAATG | GTGCTATTTGTGGCAGCGG |

**Table S2. The sequences of siRNAs, ASO and miRNA mimics**

| Gene | Targeted sequence |
| --- | --- |
| Negative control | UUCUCCGAACGUGUCACGUTT |
| Si-circRPPH1-1 | CGGGGAGGGAAGCUCAUCA |
| Si-circRPPH1-2 | AGCUUCGGGGAGGGAAGCU |
| Si-METTL3 | GGAGAUCCUAGAGCUAUUA |
| Si-METTL14 | GGCUAAAGGAUGAGUUAAU |
| Si-YTHDC1 | UGGAUUUGCAGGCGUGAAUUA |
| Si-IGF2BP1 | GGCUCAGUAUGGUACAGUA |
| Si-IGF2BP3 | GCUGAGAAGUCGAUUACUA |
| Si-SOX4 | UGGCGGAGAAGAAGGUGAATT |
| miR-7845-5p mimics | AAGGGACAGGGAGGGUCGUGG |
| ASO-circRPPH1-1 | GCTTCCCTCCCCGAAGCTCA |

**Supplementary reference**

1. Gan L, Lu T, Lu Y, Song H, Zhang J, Zhang K, Lu S, Wu X, Nie F, Di S, Han D, Yang F, Qin W, Wen W. Endosialin-positive CAFs promote hepatocellular carcinoma progression by suppressing CD8+ T cell infiltration. J Immunother Cancer. 2024; 12(9).
